# A rapid and visual detection assay for *Clonorchis sinensis* based on recombinase polymerase amplification and lateral flow dipstick

**DOI:** 10.1186/s13071-023-05774-5

**Published:** 2023-05-19

**Authors:** Xiaoxiao Ma, Xue Bai, Hongchang Li, Jing Ding, Huiyuan Zhang, Yangyuan Qiu, Jing Wang, Xiaolei Liu, Mingyuan Liu, Bin Tang, Ning Xu

**Affiliations:** 1 Key Laboratory of Zoonosis Research, Ministry of Education, Institute of Zoonosis, College of Veterinary Medicine, Jilin University, Changchun, 130012 Jilin China; 2 Qingdao Special Servicemen Recuperation Center of PLA Navy, Qingdao, 266071 Shandong China; 3 Jiangsu Co-Innovation Center for Prevention and Control of Important Animal Infectious Diseases and Zoonoses, Yangzhou, 225009 Jiangsu China

**Keywords:** *Clonorchis sinensis*, *COX1*, RPA, LFD, Visual detection, Faecal sample

## Abstract

**Background:**

Fish-borne zoonotic clonorchiasis, caused by *Clonorchis sinensis*, is an emerging public health problem in several countries with more than 15 million people infected globally. However, a lack of accurate point-of-care (POC) diagnostic tests in resource-limited areas is still a critical barrier to effective treatment and control of clonorchiasis. The development of the recombinase polymerase amplification(RPA) assay, a POC diagnostic test based on the amplification of pathogen DNA, has provided a new, simple and inexpensive tool for disease detection with high sensitivity and specificity.

**Methods:**

A novel RPA method was developed based on specific primers and probes, and combined with the dipstick, to allow for the rapid and intuitive detection of *C. sinensis* through the amplification of the mitochondrial cytochrome c oxidase subunit 1 (*COX1*) gene. The lower limit of detection for the combined RPA/lateral flow dipstick (RPA-LFD) assay was evaluated using dilutions of the target DNA sequence. Cross-reactivity was evaluated using genomic DNA from 10 additional control parasites. Forty human clinical stool samples were tested to verify its performance.

**Results:**

The evaluated primers designed from the *C. sinensis* *COX1* region can be used to detect adult worms, metacercariae, and eggs at 39 °C within 20 min, and the results can be visually observed using the LFD. The detection limit of pathogen genomic DNA was as low as 10 fg, and the number of metacercaria(e) in fish and egg(s) in faeces were both as low as one. This improved the sensitivity of low-infection detection tremendously. The test is species-specific, and no other related control parasites were detected. In human stool samples with eggs per gram (EPG) > 50, the RPA-LFD assay was performed consistent with conventional Kato-Katz (KK) and PCR methods.

**Conclusion:**

The established RPA-LFD assay provides a powerful tool for the diagnosis and epidemiological survey of *C. sinensis* from human and animal samples, and has important implications for the effective control of clonorchiasis.

**Graphical Abstract:**

**Figure:**
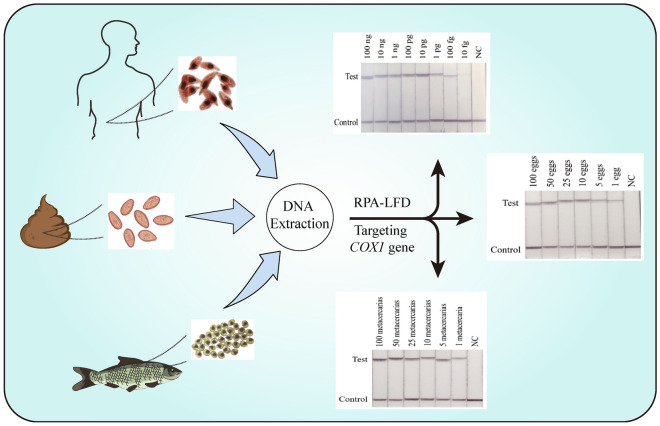

## Background

Clonorchiasis, one of the most important foodborne parasitic zoonoses, is caused by consuming freshwater fish containing infective metacercariae. Mammals susceptible to infection include canines, felines and swine, as well as humans [1]. It is estimated that approximately 15 million people are infected with *Clonorchis sinensis*, where infections are concentrated in East Asia, including Korea, China and Vietnam [2, 3]. Although the pathogen surveillance program implemented by health departments is of great significance in the prevention and control of clonorchiasis, clonorchiasis remains an uncontrolled disease in some regions due to the lack of an accurate and sensitive test, especially with low-intensity infections [4]. Developing a simple, rapid and accurate diagnostic method for detecting *C. sinensis* infection is of great importance to effectively controlling and monitoring the disease.

Traditionally, the detection of *C. sinensis* was routinely carried out, with variable sensitivity and specificity, by identifying eggs in the faeces or adult worms in the hepatobiliary duct using microscopy or autopsy [5]. Aetiological examination is still the gold standard, but it is time-consuming, requires professional operation, and has a high false positive rate for low-intensity infections [6]. Immunologic methods, such as enzyme-linked immunosorbent assay (ELISA), immunochromatographic test (ICT) and time-resolved fluorescein immunoassay (TRFIA) are very sensitive and are useful for epidemiological studies and large-scale disease surveillance [7–9]. However, these methods are prone to high cross-reactions and false positives, and it is difficult to distinguish between current and previous infections. Molecular methods could provide an effective alternative to the aforementioned methods and are of greater sensitivity and specificity [10]. Diagnostic methods such as conventional polymerase chain reaction (PCR), real-time PCR, nested PCR, and multi-PCR methods have been developed to detect *C. sinensis* DNA in several samples [11]. Although PCR-based assays are highly sensitive and can detect low parasite burdens, the need for specific tools such as high-precision thermal cyclers has limited its application in resource-limited settings [12].

Recombinase polymerase amplification combined with lateral flow dipstick (RPA-LFD), an isothermal DNA amplification technology, mainly depends on recombinase (a single-stranded binding protein [SSB]), DNA polymerase and lateral flow dipstick [13]. The amplified products can be easily read, which makes the endpoint analysis more flexible and feasible in low-resource soundings [14]. Moreover, DNA amplification can be achieved by using closely spaced opposing primers within 30 min at temperatures between 30 and 45 °C. Recently, several studies have shown that RPA-LFD can be successfully applied to the detection of parasites with high sensitivity, specificity and convenient results, including *Schistosoma japonicum*, *Trichinella* spp. and *Babesia orientalis* [15–17]. However, there is still no RPA-LFD test available for the detection of *C. sinensis*.

The mitochondrial cytochrome c oxidase subunit 1 (*COX1*) gene is an accurate genetic marker for the identification of many parasites [4, 17]. Hence, the objective of the present study was to develop, using *COX1* as the genetic marker, a rapid and visual detection test using RPA-LFD.

## Methods

### Sample collection

*Clonorchis sinensis* metacercariae were collected from naturally infected *Pseudorasbora parva* in the endemic area of Changchun, Jilin province, China. Muscular tissue was digested with artificial digestive juice (1% pepsin-hydrochloric acid, Aladdin, China) [3]. Beagles (6–8 weeks old), purchased from the Experimental Animal Center of Jilin University, were orally infected with 500 viable metacercariae. Twenty days post-infection, faeces samples were collected from each beagle for egg examination. The adult worms of *C. sinensis* were separated from the bile ducts of infected beagles at 45 days post-infection, and washed three times with phosphate-buffered saline (PBS). All animal experiments in this study were approved by the Ethical Committee of Jilin University affiliated with the Provincial Animal Health Committee, China (ethical clearance number 2021530).

Several related and common parasites were included as control samples while assessing the specificity of the RPA assay, including *Opisthorchis viverrini*, *Metorchis orientalis*, *Schistosoma japonicum*, *Fasciola hepatica*, *Taenia solium*, *Taenia saginata*, *Taenia asiatica*, *Ascaris lumbricoides*, *Ancylostoma duodenale* and *Cryptosporidium parvum*. The adult worm of *F. hepatica* was kindly provided by Chunren Wang (Heilongjiang Bayi Agricultural University); genomic DNA from *C. parvum* was presented by Guan Zhu (Jilin University); other parasites were obtained from stocks in our institute. All parasite samples were preserved in 70% ethanol and kept at −80 °C until further use. For the analysis of the RPA assay, stool samples were randomly selected from the residents of Fuyu city, Jilin Province, where clonorchiasis is endemic. All experiments were approved by the Ethical Committee of Jilin University, China (ethical clearance number 2021703).

### DNA extraction

Total genomic DNA of *C. sinensis* adults, metacercariae, eggs and other control samples was extracted individually using the TIANamp Genomic DNA Kit (Tiangen, Beijing, China) following the manufacturer’s instructions. DNA from human stool samples was extracted using the QIAamp Tissue Kit Spin Columns (Qiagen, Hilden, Germany) following the manufacturer's protocols. The integrity of all DNA samples was validated by the successful amplification of the ribosome internal transcribed spacer (ITS) region using published primers NC2 and NC5 [18].

### Primers and probe design

The highly specific *COX1* gene of *C. sinensis* was selected as the target sequence, and its reference sequence was downloaded from the GenBank database (GenBank no. MF287785). Primers and the probe for the RPA assay specific to the *COX1* gene were designed according to the TwistDX guidelines (http://www.twistdx.co.uk). The primer dimer, hairpin formation and other biophysical properties were analysed with OLIGO Primer Analysis software, and the nucleotide database of the National Center for Biotechnology Information (NCBI) was blasted to verify the lack of sequence homology with other related species. Finally, a 209-base-pair (bp) fragment of the *COX1* gene was selected, and the length was consistent with the requirement of the RPA method. To be visualized by the lateral flow detection system, biotin was added to the 5′ end of the reverse primer, and the probe was labelled with a 5′-fluorescein amidite (FAM); a tetrahydrofuran (THF) residue was inserted between the 30th and 31st bases, and a C3 spacer (SpC3) at the 3′ end (Table 1).

**Table 1:** RPA-LFD primers and probe used in the present study

Primers	Length	Sequence(5′–3′)
RPA-F	32	ATATGCTTGCTGGAACTCGGGAGCGTCTATGA
RPA-R	32	TACAGAAATAACAACCGTCCTAAACGACCCTA
Lateral flow reverse primer	32	Biotin-TACAGAAATAACAACCGTCCTAAACGACCCTA
Lateral flow probe	46	FAM-CTTTCTGCTTCTGTAATTGATGCCTTGTTT-THF-CATGATACTTGGTTTG-C3 Spacer

### RPA reaction and lateral flow reading

The RPA reaction system was established using a TwistAmp nfo kit (TwistDX Ltd., Cambridge, UK) according to the manufacturer’s instructions. Each RPA reaction was performed in a PCR tube with a total volume of 47.5 μl containing 25 μl rehydration buffer, 16.7 μl ddH_2_O, 2.1 μl of each forward and reverse primer (final concentration: 420 nM), 2 μl DNA template and 0.6 μl probe (final concentration: 120 nM). After shaking slightly, 2.5 μl (280 mM) of magnesium acetate was added to the lid of the tube and mixed in. Reactions of the RPA-LFD assay were run for 20 min at 39 °C in a metal heater. For the detection of the RPA amplicon directly, 2 μl of the amplification product was diluted to 100 μl with the provided dipstick assay buffer. Then, 50 μl of the diluted sample was transferred to the sample pad of a lateral flow strip (Milenia Biotec, Germany) and incubated for 5 min at room temperature.

Positive DNA amplification was observed with the naked eye as the appearance of both the test line and control line, where negative results only show a control line in the below part of the strip. The amplification temperature and reaction duration were important factors affecting the RPA-LFD assay. To select the optimal conditions, the RPA assay was run at different reaction temperatures (20 °C, 25 °C, 30 °C, 39 °C, 45 °C and 50 °C) and for different time periods (5, 10, 15, 20, 25 and 30 min).

### Sensitivity of the RPA-LFD assay

The sensitivity of the RPA-LFD assay was determined by amplifying 10-fold serial dilutions of genomic DNA of *C. sinensis* adult worms at quantities from 100 ng to 10 fg. The detection limit was compared with that of commonly used PCR assays. In addition, to test the detection line of eggs and metacercariae, the total genomic DNA of purified and quantity-known egg(s) (1–100) and metacercaria(e) (1–100) was extracted and amplified using the RPA assay. Each of the reactions was performed in triplicate according to the pre-determined optimal conditions. The PCR primers, CS1 (5′-CGAGGGTCGGCTTATAAAC-3′) and CS2 (5′-GGAAAGTTAAGCACCGACC-3′) were used to amplify the partial internal transcribed spacer 2 (ITS2) gene with a length of 350 bp in *C. sinensis* [19]. The amplification conditions were as follows: 94 °C for 3 min; three-step cycling by denaturation at 94 °C for 1 min, annealing at 62 °C for 1 min and extension at 72 °C for 1 min, and the number of cycles conducted was 40; a final extension step was carried out at 72 °C for 10 min. The amplification products were detected by agarose gel electrophoresis.

### Specificity of the RPA-LFD assay

The specificity of the RPA assay was evaluated by cross-reaction tests using a range of DNA templates from other parasites (see details above). The concentration of the genomic DNA of each sample was measured by spectrophotometer, diluted to a final concentration (10 ng/μl), and then added to the RPA assay. All these templates were tested in triplicate under the optimal conditions determined for the RPA-LFD assay.

### Egg spiking

To demonstrate the feasibility of estimating infection intensities, adult worms of *C. sinensis* were cultured in vitro at 37 °C, 5% CO_2_ for 12 h. Then, the culture medium was collected, and the eggs were enriched after centrifugation. The harvested *C. sinensis* eggs were counted and added to 200 mg fluke egg-negative human faeces in a range of numbers [i.e., 5000, 1000, 500, 100, 50, 25, 10, 5, and 1 egg(s)]. The faecal samples were collected from fluke-free humans based on age and living habits. Each sample was tested 10 times using the Kato-Katz (KK) and PCR methods, to confirm that the faeces were fluke-free [4]. To extract DNA from stool samples, 200 mg of faeces was washed three times with 1 ml PBS and centrifuged at 9000 rpm for 10 min, and the pellet was then resuspended with 200 μl 2% polyvinylpolypyrrolidone solution and heated at 100 °C for 10 min. Followed by treating at 55 °C for 2 h with sodium dodecyl sulfate and protease K, the eluting process was performed by adding 80 μl buffer solution to a column of QIAamp Tissue Kit (Qiagen, Hilden, Germany) according to the manufacturer’s instructions. For each egg-spiked stool sample, the total genomic DNA was tested with PCR and RPA-LFD individually.

### Applicability of the RPA-LFD assay

The applicability of the RPA-LFD assay in the detection of *C. sinensis* was evaluated using 40 human stool samples, which were randomly selected from the residents of Fuyu city (Jilin Province China) where clonorchiasis is endemic. Each faecal sample was divided into three subsamples: (1) one part was tested with the KK technique, (2) one part with the PCR method (3) and one part with the RPA-LFD assay. Total DNA was extracted from 200 mg of all 40 samples using the method described above, and the sample was considered positive if either the KK smear or PCR detection were positive [4].

## Results

### Optimization of the RPA-LFD assay

**Fig. 1:**
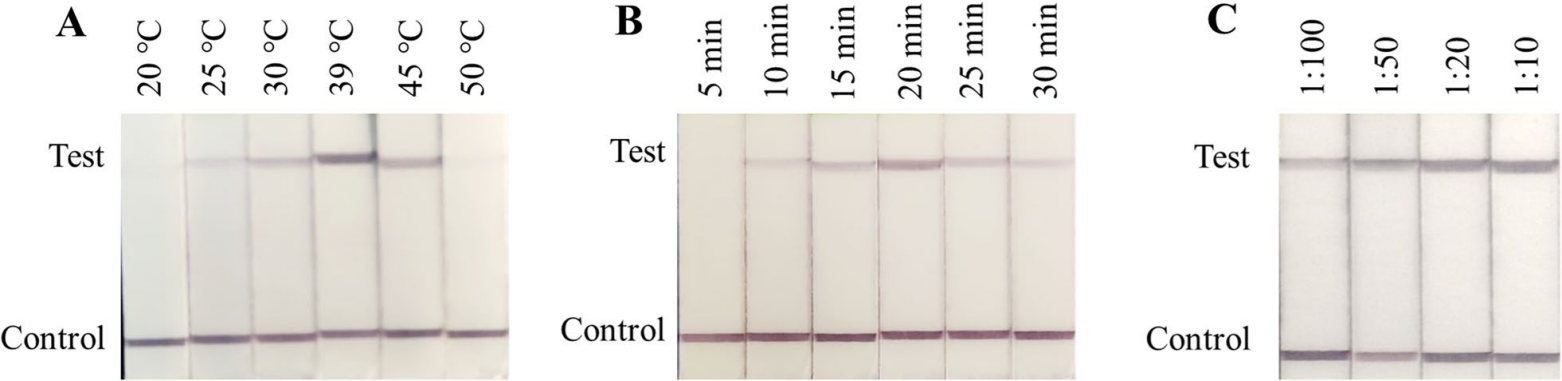
Optimization of the RPA-LFD assay. **A** Optimization of the amplification temperature. The RPA-LFD assay results are positive at temperatures from 25 °C to 45 °C. **B** Optimization of the amplification duration. The PCR-LFD assay results are positive for 5, 10, 15, 20, 25 and 30 min duration. **C** Optimization of the amplification product dilution. The RPA-LFD assay results are positive at amplification product dilutions of 1:10, 1:20, 1:50, and 1:100

The RPA-LFD assay could function at a wide range of temperatures with different amplification signals. The test signals gradually became brighter with an increase in amplification temperature. The clearest amplification signal was seen at 39 °C, followed by 45 °C; therefore, 39 °C was set as the optimum amplification temperature. No amplification signals were detected when the reaction was performed at 20 °C or 50 °C (Fig. 1A). Amplification bands were visible in the time range from 10 to 30 min, where amplification for 20 min showed the clearest bands (Fig. 1B). Amplification products were diluted with running buffer at ratios of 1:10, 1:20, 1:50, and 1:100. Test bands were observed at all four dilutions, and a visible test band was observed at 1:10, 1:20 and 1:50 dilutions. A dilution of 1:50 was determined as the optimal dilution (Fig. 1C).

### Specificity and sensitivity analysis of RPA-LFD

**Fig. 2:**
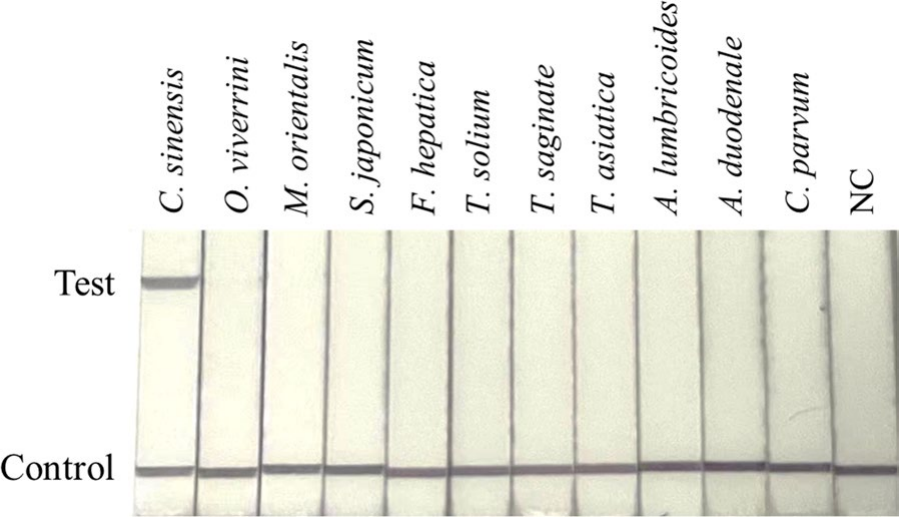
Specificity of the RPA-LFD assay. The LFD-RPA assay detects *C. sinensis* exclusively and exhibits no cross-reactivity with genomic DNA from other parasites. *NC* negative control, containing no DNA

To evaluate the specificity of RPA-LFD, the RPA amplification was performed under the optimal conditions by using the purified genomic DNAs of various ‘heterologous control samples’, namely, *O. viverrini*, *M. orientalis*, *S. japonicum*, *F. hepatica*, *T. solium*, *T. saginata*, *T. asiatica*, *A. lumbricoides*, *A. duodenale* and *C. parvum*. There was no cross-reaction with any of the heterologous control samples, suggesting high specificity of the RPA assay in distinguishing *C. sinensis* from other pathogens by comparing the visualized control and test lines (Fig. 2).

**Fig. 3:**
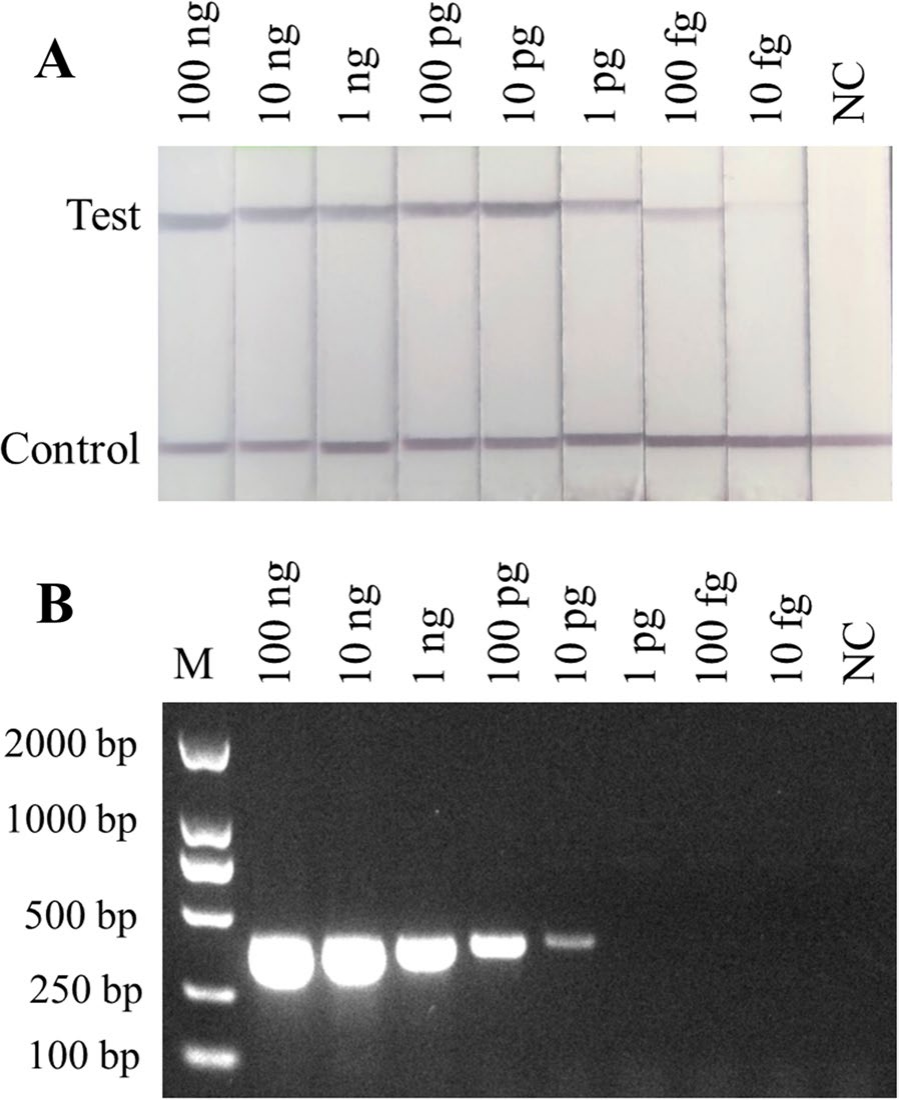
Sensitivity of the RPA-LFD assay and PCR for the detection of *C. sinensis* genomic DNA. **A** Sensitivity of the RPA-LFD assay. Genomic DNA of *C. sinensis* at quantities from 100 ng to 10 fg was used to determine the minimum detection concentration in the LFD-RPA assay. **B** PCR with reported primers Cs1 and Cs2 with DNA quantities from 100 ng to 10 fg. *NC* negative control, containing no DNA

**Fig. 4:**
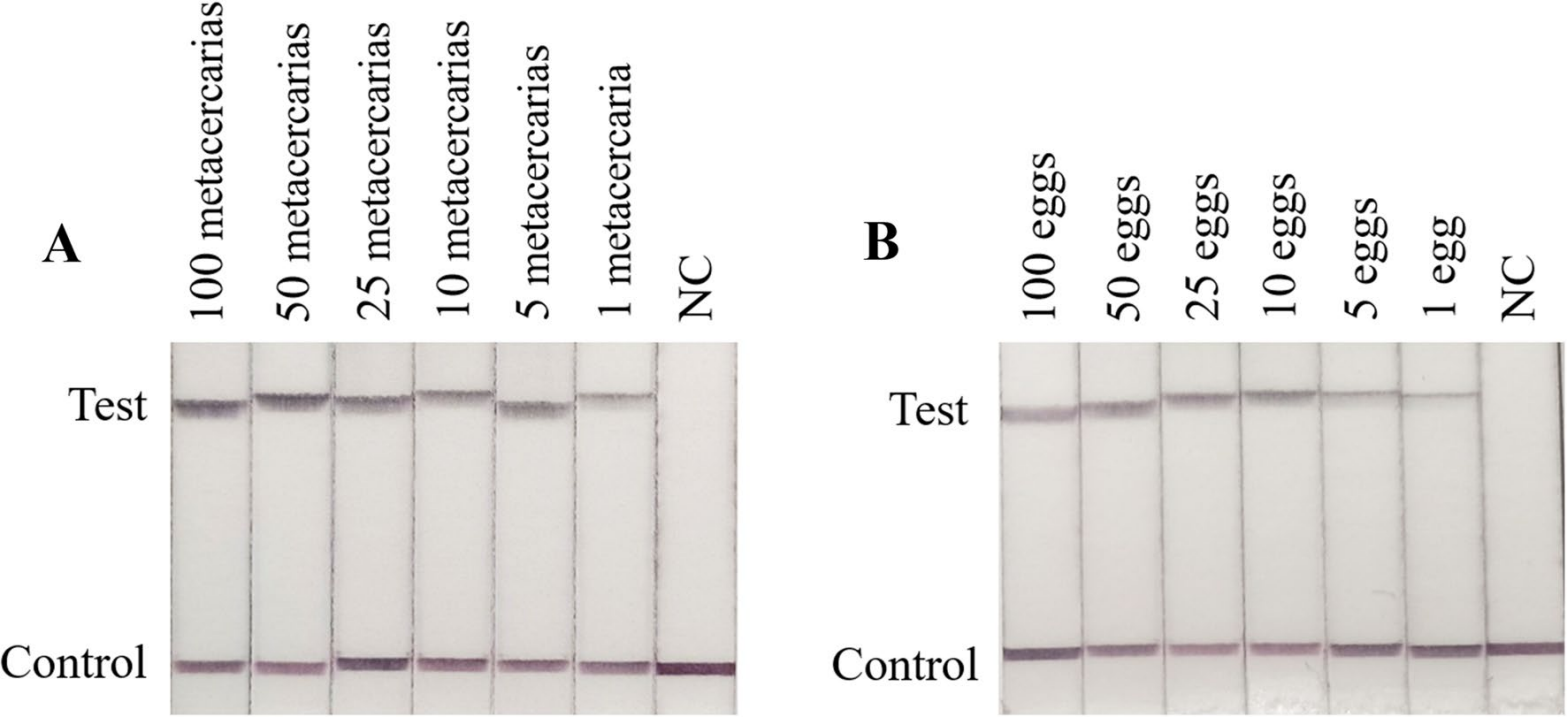
The detection limit line of the purified *C. sinensis* metacercaria(s) and egg(s). **A** Sensitivity of the RPA-LFD assay for the detection of purified *C. sinensis* metacercaria(s). The known number of metacercaria(s) from 1–500 *NC* negative control, containing no metacercaria. **B** Sensitivity of the RPA-LFD assay for the detection of purified *C. sinensis* egg(s). The known number of egg(s) from 1 to 500. *NC* negative control, containing no egg

The sensitivity of the RPA-LFD assay was tested and compared with the PCR tests including *C. sinensis* adult worm DNA (at quantities from 100 ng to 10 fg), and the purified egg(s) and metacercaria(s) [metacercarias (100–1) and eggs (100–1)]. The results showed that the detection limit of the RPA-LFD assay was 10 fg (Fig. 3A), while that of the conventional PCR test was 10 pg (Fig. 3B). The detection limits of purified egg(s) and metacercaria(e) were both as low as 1 (Fig. 4A, B).

### Egg detection limit from spiked samples

**Fig. 5:**
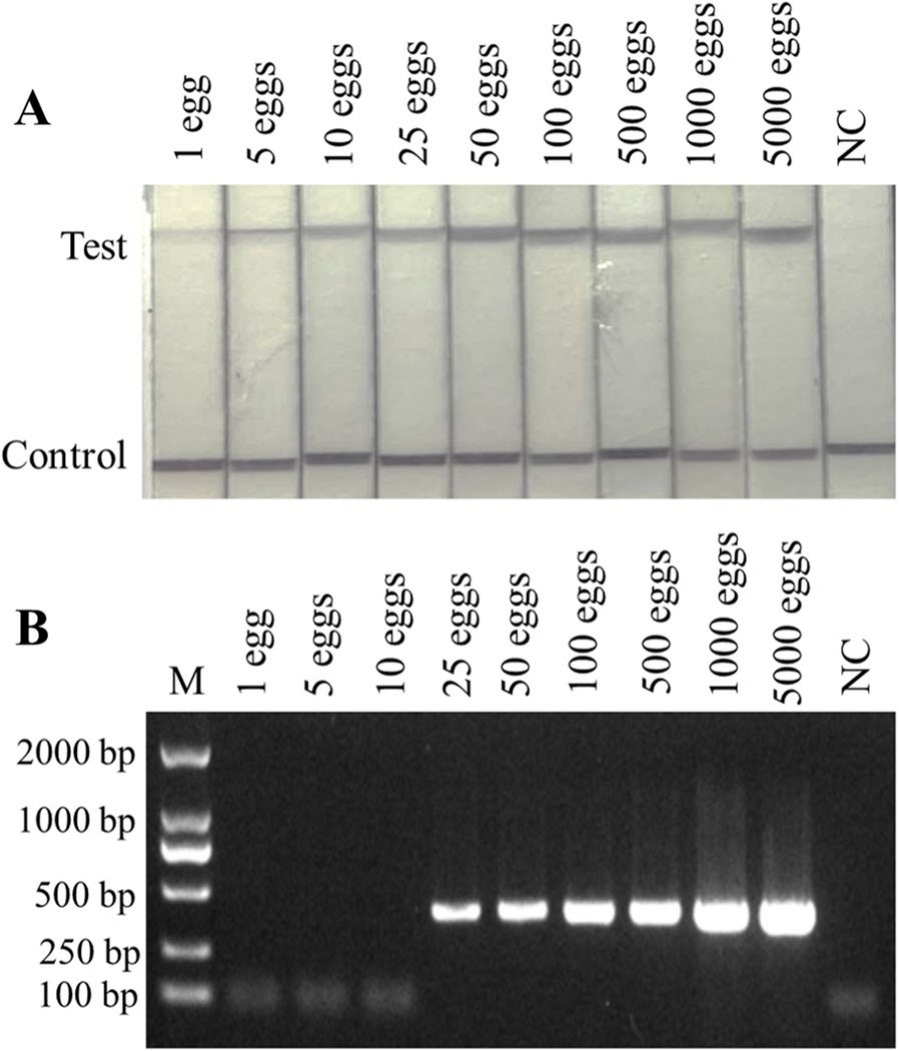
Sensitivity of the RPA-LFD assay for egg-spiked samples. **A** Sensitivity of the RPA-LFD assay in experimentally spiked samples. Sensitivity of the RPA-LFD assay for the detection of *C. sinensis* eggs in faeces experimentally spiked with a known number of eggs from 1 egg to 5000 eggs. **B** PCR with reported primers Cs1 and Cs2 for the detection of *C. sinensis* eggs in faeces experimentally spiked with a known number of eggs from 1 egg to 5000 eggs. *NC* negative control, containing no egg

To demonstrate the feasibility of estimating infection intensity, we analysed the DNA extracted from stools spiked with *C. sinensis* eggs at different levels. The results of the egg detection limit test showed positive amplification from 5000 eggs down to one egg using the RPA-LFD, which highlighted the likelihood that the RPA-LFD assay can detect an infected individual with low-intensity infection (Fig. 5A). By comparison, although the conventional PCR showed a good detection effect, with a spiked egg detection limit above 25 eggs, it was still inferior to RPA-LFD assay (Fig. 5B).

### Comparative profiling of microscopy, PCR and RPA-LFD on clinical samples

The diagnostic efficiency of the RPA assay was evaluated using 40 human stool samples, which were randomly selected from the residents of Fuyu County (Jilin Province, China), where clonorchiasis is endemic. The sample was divided into four groups based on the age distribution. PCR and RPA-LFD had a consistent positive rate of 35% (14/40) with the eggs per gram (EPG) > 50. Among the 14 positive cases, there were 10 positive cases (B7, C3, C5, C7, D2, D4, D5, D6, D8, D9) confirmed by all three detection methods with the EPG > 50, and four cases (A8, B3, B9, C1) tested positive with the PCR and RPA-LFD test but were microscopy-negative with the EPG > 50 (Table 2).

**Table 2:** Comparative microscopy, PCR and RPA-LFD for detection of *Clonorchis sinensis* in 40 human stool samples

Case code	Age	EPG	Microscopy	PCR	RPA-LFD
A1	20–30	0	**−**	**−**	**−**
A2	20–30	0	**−**	**−**	**−**
A3	20–30	0	**−**	**−**	**−**
A4	20–30	0	**−**	**−**	**−**
A5	20–30	0	**−**	**−**	**−**
A6	20–30	0	**−**	**−**	**−**
A7	20–30	0	**−**	**−**	**−**
A8	20–30	< 50	**−**	**+**	**+**
A9	20–30	0	**−**	**−**	**−**
A10	20–30	0	**−**	**−**	**−**
B1	30–40	0	**−**	**−**	**−**
B2	30–40	0	**−**	**−**	**−**
B3	30–40	< 50	**−**	**+**	**+**
B4	30–40	0	**−**	**−**	**−**
B5	30–40	0	**−**	**−**	**−**
B6	30–40	0	**−**	**−**	**−**
B7	30–40	50–100	**+**	**+**	**+**
B8	30–40	0	**−**	**−**	**−**
B9	30–40	< 50	**−**	**+**	**+**
B10	30–40	0	**−**	**−**	**−**
C1	40–50	< 50	**−**	**+**	**+**
C2	40–50	0	**−**	**−**	**−**
C3	40–50	> 100	**+**	**+**	**+**
C4	40–50	0	**−**	**−**	**−**
C5	40–50	50–100	**+**	**+**	**+**
C6	40–50	0	**−**	**−**	**−**
C7	40–50	50–100	**+**	**+**	**+**
C8	40–50	0	**−**	**−**	**−**
C9	40–50	0	**−**	**−**	**−**
C10	40–50	0	**−**	**−**	**−**
D1	> 50	0	**−**	**−**	**−**
D2	> 50	> 100	**+**	**+**	**+**
D3	> 50	0	**−**	**−**	**−**
D4	> 50	50–100	**+**	**+**	**+**
D5	> 50	50–100	**+**	**+**	**+**
D6	> 50	> 100	**+**	**+**	**+**
D7	> 50	0	**−**	**−**	**−**
D8	> 50	50–100	**+**	**+**	**+**
D9	> 50	> 100	**+**	**+**	**+**
D10	> 50	0	**−**	**−**	**−**

## Discussion

Clonorchiasis, an emerging public health problem, can lead not only to mechanical irritation of the bile ducts, but also to chemical impairment including biliary epithelial hyperplasia and periductal fibrosis, and can even facilitate the development of cholangiocarcinoma [11, 20]. In recent decades, after extensive efforts to prevent and control clonorchiasis, the incidence of this disease has decreased significantly in China [1]. However, there are still some risk factors, such as eating habits and the lack of an effective surveillance system, resulting in the transmission of clonorchiasis in low-prevalence areas in China [20]. In addition, low-intensity infection of *C. sinensis* remain a serious and urgent problem and are also the main inhibitory factor in the prevention and control of clonorchiasis. Despite the existence of a variety of sensitive and specific detection tools, conventional faecal examination is still the gold standard, although there are some drawbacks such as false negatives and the similarity of egg morphology with other flukes [2, 21]. Mitochondrial (mt) sequences are widely used for the classification of flukes at higher taxonomic levels due to its strict maternal inheritance, rapid evolution and relatively conservative genome [22]. Studies indicate that the mitochondrial *COX1* gene can serve as an accurate genetic marker for the identification of many parasites using isothermal amplification methods. These tests can overcome traditional drawbacks, and their features greatly simplify the implementation of these methods in point-of-care (POC) diagnosis [4, 17, 23, 24].

Recently, the RPA assay technique has been successfully applied for the detection of pathogens, including bacteria, viruses, and parasites [25–27]. Compared with conventional PCR, RPA has the advantages of high sensitivity, fast detection time, and instant visual observation, which make it well-suited for field application [28, 29]. In this study, we developed an RPA-LFD assay targeting the *C. sinensis* *COX1* gene and evaluated its diagnostic efficacy and specificity at three developmental stages (adult worm, metacercariae and eggs). The selected RPA primers are located in a highly differentiated region of the *COX1* gene, allowing them to distinguish *C. sinensis* from other tested heterologous control trematodes, including *O. viverrini*, *M. orientalis*, *S. japonicum*, *F. hepatica*, *T. solium*, *T. saginata*, *T. asiatica*, *A. lumbricoides*, *A. duodenale* and *C. parvum*. The infection rate of *C. sinensis* in its hosts can be quite low, so the sensitivity of the assay is extremely important [30]. Fortunately, the application of fluorescently labelled primers improved the detection efficiency: the reverse primers and probes were labelled with biotin and FAM, respectively, resulting in double-labelled RPA products being caught on lateral flow strips with gold-labelled anti-FAM antibodies [31]. With the labelled primers, the established RPA-LFD assay is sensitive enough to detect as little as 10 fg of *C. sinensis* genomic DNA, which is 1000 times as sensitive as conventional PCR.

It is important to note that there are no previous reports of an RPA-LFD test for *C. sinensis* from human stool samples [32]. At present, only a few isothermal amplification methods, mainly focused on loop-mediated isothermal amplification (LAMP), have been applied for the detection of *C. sinensis* DNA. LAMP assays applied to human faecal samples showed 97.1% sensitivity and 100% specificity in stool samples with more than 100 EPG of faeces [4]. Two other studies detected *C. sinensis* from its intermediate hosts, freshwater snails and fish, and proved to be 100 and 1000 times as sensitive as a PCR test, respectively [23, 33]. Although these studies showed good performance, the results were not visible in real time, adding to the workload, cost and duration of the test.

The minimum detection limit of the established visual RPA-LFD assay was as low as one egg in 200 mg of stool experimentally spiked with a series of known numbers of *C. sinensis* eggs. With the low detection limit, our RPA-LFD assay achieved sensitivity as high as 100% (14/14), similar to that of the conventional PCR test. Four samples tested positive using RPA-LFD and PCR but negative with the KK assay at an EPG < 50. The number of eggs in stool samples is often small and the distribution is not uniform; thus, sampling could miss the eggs, leading to false negative results, possibly explaining the false negative of the KK smear test (10/14). Given the potentially low number of eggs in stool samples from patients with subclinical disease, the combined use of multiple detection methods can effectively improve the accuracy of detection [4, 12].

The RPA-LFD assay established in the present study has several advantages: (1) it is easy to perform, and the use of freeze-dried reagents simplifies the workload; (2) it has a faster reaction time, less than 20 min for RPA-LFD versus several hours for PCR; (3) it requires fewer instruments and equipment, only a constant-temperature instrument is needed; (4) results are easier to visualize, and amplification products can be read with the naked eye and interpreted by untrained personnel. However, the cost of the RPA reagents and kits is high relative to the cost of conventional PCR analysis. Also, the method is limited to laboratory use due to the need for sterile genomic DNA extraction. It is therefore necessary to develop a simple DNA extraction method to promote field application [34].

## Conclusion

We developed a novel RPA-LFD assay that allows for the rapid and sensitive detection of *C. sinensis* genomic DNA. This assay is faster and easier to perform and assess than conventional methods, and performed well in clinical tests. It is also more applicable to field laboratories than conventional PCR methods. This technique has broad application prospects in clinical and epidemiological studies and can aid in the surveillance and control of clonorchiasis.

## Data Availability

The dataset supporting the findings of this article is included within the article.

## Abbreviations

RPA: Recombinase polymerase amplification
RPA-LFD: Recombinase polymerase amplification combined with lateral flow dipstick
POC: Point of care
*COX1*: Cytochrome c oxidase subunit 1
ELISA: Enzyme-linked immunosorbent assay
ICT: Immunochromatographic test
TRFIA: Time-resolved fluorescence immunoassay
SSB: Single-stranded binding protein
ITS: Internal transcribed spacer
FAM: Fluorescein amidite
THF: Tetrahydrofuran
SpC3: C3 spacer
KK: Kato-Katz
EPG: Eggs per gram
